# A sudden decrease in voice volume: A rare manifestation of spontaneous pneumomediastinum

**DOI:** 10.1002/jgf2.426

**Published:** 2021-02-16

**Authors:** Seigo Urushidani, Akira Kuriyama

**Affiliations:** ^1^ Emergency and Critical Care Center Kurashiki Central Hospital Okayama Japan

**Keywords:** chest X‐ray, computed tomography, dysphonia, mediastinal emphysema, pneumomediastinum

## Abstract

Spontaneous pneumomediastinum (SPM) is a rare condition characterized by free air in the mediastinum that primarily affects young individuals between the ages of 10 and 30 years. The most frequent symptoms of SPM are chest pain and dyspnea. However, a decrease in voice volume without a change of tone is a rare presentation. SPM is generally a benign and self‐limiting condition, but it can occasionally cause tension pneumothorax. If a young patient presents with a sudden decrease in voice volume without a change of tone, SPM should be considered as a possible diagnosis.

## Graphical abstract

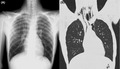

## 1 INTRODUCTION

Spontaneous pneumomediastinum (SPM) is a rare condition characterized by free air in the mediastinum.^1^, ^2^, ^3^ It primarily affects young individuals between the ages of 10 and 30 years.^2^, ^3^ The most common symptoms are chest pain and dyspnea, but dysphonia is infrequent.^2^ A decrease in voice volume without a change in tone has not been reported previously. Here, we present a case of an adolescent boy who experienced a sudden decrease in the volume of his voice without a change of tone and was diagnosed with SPM.

## 2 CASE REPORT

A previously healthy 14‐year‐old boy presented to the emergency department (ED) with a sudden decrease in the volume of his voice without a change of tone. His parents noticed the changes and brought him to our ED. In addition, his friends noticed that the volume of his voice had suddenly decreased while cheering for his volleyball team. He also reported experiencing slight chest discomfort and mild pain in front of his neck. He experienced no cough, dyspnea, nausea, sore throat, or abdominal pain and had no symptoms of respiratory tract infection or history of respiratory disease.

His vital signs on arrival were as follows: blood pressure 127/74 mmHg, pulse 81 beats/minute, respiratory rate 16 breaths/minute, and temperature 37.1°C, and he was alert and fully conscious. Oxygen saturation of the peripheral artery was 98%. His voice was small, but not hoarse. On examination, his pharynx was not inflamed and palatine tonsils were not swollen. There was no thyroid goiter, and he had palpable crepitations on the right side of his neck and around the right clavicle. His respiratory sounds and heart sounds were normal. Hamman's sign, which is a crunching, rasping sound that synchronizes with the heartbeat, was not detected.

**Figure 1:**
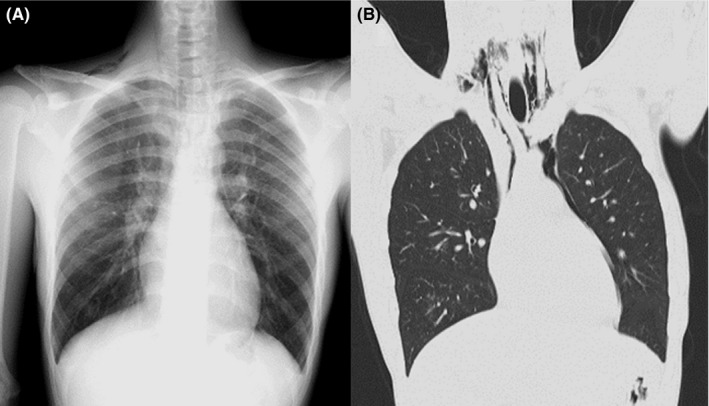
(A) Chest X‐ray showing emphysema above the right clavicle and in the mediastinum. (B) Chest computed tomography shows emphysema in the mediastinum and behind the right clavicle

Chest X‐ray and computed tomography revealed emphysema on his neck, mediastinum, and around the right clavicle (Figure 1A and B). Based on the clinical presentation and the imaging findings, he was diagnosed with SPM. He was advised not to engage in activities that might increase his intrathoracic pressure such as blowing his nose or shouting. At a follow‐up visit 10 days later, his voice was louder, the palpable crepitations had disappeared, and chest X‐ray revealed that the emphysema had disappeared.

## 3 DISCUSSION

A healthy adolescent boy presented with a sudden decrease in the volume of his voice without a change of tone. He was diagnosed with SPM based on palpable crepitations on physical examination and imaging findings. Triggers such as cough are detected in approximately 50% of cases.^2^, ^3^ The causative mechanism of SPM is believed to be increased intra‐alveolar pressure due to excessive inspiration, forced expiration, or cough, which breaks the alveolar bases; air thus flows into the connective tissue of the lung and then disseminates from the lung to the mediastinum along the vessels due to the pressure gradient.^4^ As our patient had no concomitant respiratory conditions, cheering for his volleyball team may have caused an increased intra‐alveolar pressure, leading to SPM.

**Figure 2:**
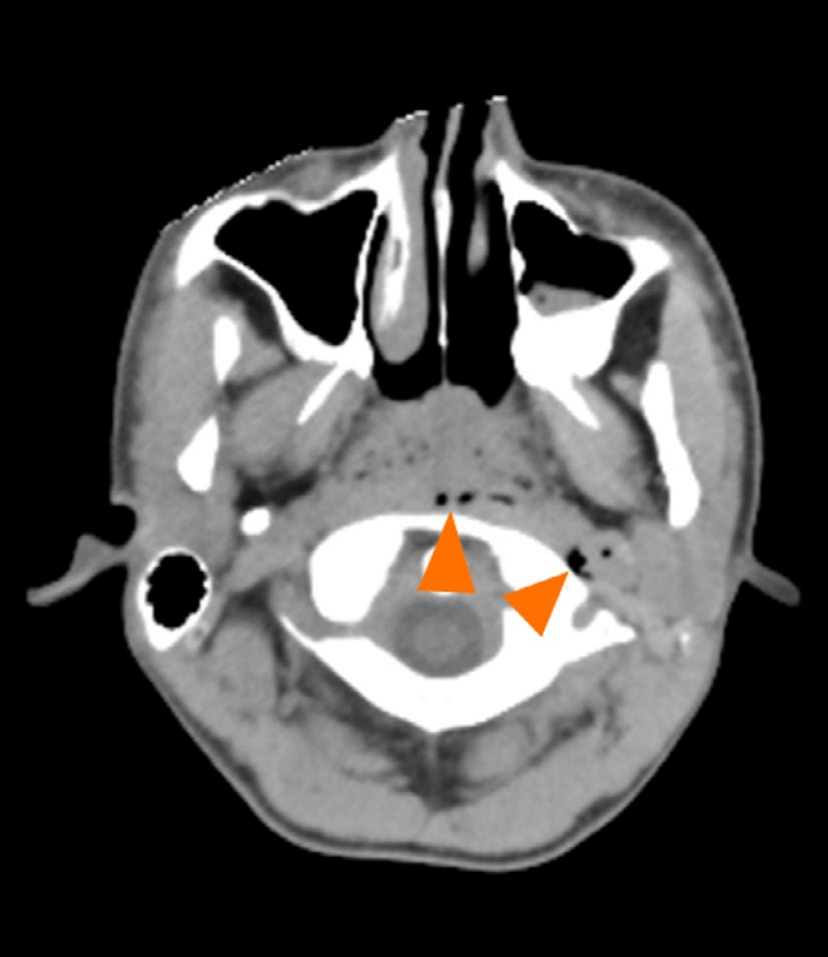
Head computed tomography shows emphysema in the nasopharynx

Although voice changes in SPM may include hoarseness, a high‐pitched tone, and rhinolalia,^5^, ^6^, ^7^ SPM presenting as a sudden decrease in voice volume without a change of the tone has not been reported previously. We hypothesized that the patient's sudden decrease in voice volume was caused by emphysema that extended to the nasopharynx (Figure 2), disturbing the passage of air and voice sounds, and that his chest discomfort and neck pain resulted in shallow breathing.

Dysphonia is defined as difficulty or pain in speaking. Approximately 1% of the population complains of dysphonia and approximately 50% of such cases are caused by acute laryngitis.^8^ Especially in children, vocal nodules are the most common cause of dysphonia.^9^ Acute inflammation of the larynx or lesion of the vocal nodules affects the vocal cord directly, causing dysphonia. However, our patient had subcutaneous or submucosal air due to SPM, which did not affect the vocal cord directly. Thus, it is suspected that our patient was not aware of the decrease in the volume of his voice, which was noticed by the people around him, or did not complain of dysphonia. SPM is a generally benign condition and usually improves over several days, but it can occasionally cause tension pneumothorax.^10^ A decrease in the voice volume without a change of tone is a rare presentation and therefore difficult to be diagnosed by treating physicians. In our case, the sudden onset of the symptoms and the presence of subcutaneous emphysema alerted us to the diagnosis. Careful history taking and examination is crucial in evaluating young patients with decreasing voice volume without tone change.

## 4 CONCLUSIONS

A sudden decrease in voice volume without a change of tone is a unique and rare symptom. If a young individual presents with a sudden decrease in voice volume without a change of tone, SPM should be considered as a possible diagnosis.

## CONFLICT OF INTEREST

All authors have no conflict of interest to declare.

## ETHICS APPROVAL

The investigation was conducted in accordance with approval of the institutional ethics committee and declaration of Helsinki.

## INFORMED CONSENT

The patient's parents have provided written informed consent, and the patient has provided written assent to the publication of this report.
